# Aligning AI affordances with critical thinking skills in Chinese EFL: a discrepancy-based needs analysis for an AI-integrated blended reading module

**DOI:** 10.3389/fpsyg.2026.1811899

**Published:** 2026-04-13

**Authors:** Anni Yang, Liyun Dong

**Affiliations:** 1 Faculty of Education, Universiti Kebangsaan Malaysia, Bangi, Malaysia; 2 School of Foreign Languages, Xiamen University of Technology, Xiamen, China

**Keywords:** artificial intelligence, blended learning, cognitive–technological alignment, critical thinking skills, EFL reading, needs analysis

## Abstract

**Introduction:**

Artificial intelligence (AI) is increasingly embedded in blended learning (BL) environments, yet its integration frequently emphasizes efficiency and performance outcomes rather than the systematic cultivation of higher-order cognition. Addressing this gap, the present study employs a discrepancy-based needs analysis to investigate how critical thinking skills (CTS) can be deliberately aligned with AI affordances in blended reading among Chinese undergraduates.

**Methods:**

Drawing on qualitative evidence from 20 Chinese EFL undergraduates, this study collected data through AI-mediated reading interactions, reflective learning logs, semi-structured interviews, and a focused literature–technology scan. A discrepancy-based needs analysis was conducted to examine the alignment between AI affordances, instructional design, and critical thinking development.

**Results:**

The findings suggest three interrelated structural misalignments between AI affordances, instructional design, and critical thinking development: (1) predominance of literal comprehension over analytical and evaluative processing, (2) limited metacognitive self-regulation, and (3) instrumental use of AI for surface-level linguistic assistance rather than structured reasoning support. Despite these patterns, learners appear ready for CTS-oriented blended learning and articulate clear expectations for explicit cognitive modelling, dialogic engagement, and adaptive AI feedback that evaluates reasoning quality rather than correctness alone.

**Discussion:**

In response to these findings, the study proposes a design-oriented Technology–CTS Needs Matrix that integrates empirical findings from participant data with literature- and theory-informed design extrapolations, systematically mapping Facione’s six CTS dimensions onto targeted AI and pedagogical affordances. By reconceptualising needs analysis as a mechanism for instructional alignment rather than deficiency identification, the study provides a theoretically integrated and empirically grounded foundation for structuring AI-mediated blended reading environments that foster critical thinking skills in EFL contexts.

## 1 Introduction

In an era of rapid technological advancement and digital transformation, critical thinking skills (CTS) are widely recognized as essential competencies in higher education (Facione, 1990; OECD, 2019; Fitriati and Williyan, 2025). In English as a Foreign Language (EFL) contexts, CTS enable learners to move beyond literal comprehension and engage in analytical interpretation, evaluative judgment, and evidence-based meaning construction across diverse texts (George and Kumar, 2024). As digital platforms and AI-mediated information ecosystems increasingly shape academic literacy practices, learners are expected not only to comprehend texts but also to critically interrogate multimodal, algorithmically curated information (Zawacki-Richter et al., 2019; Haşlaman et al., 2024). Although CTS have been shown to enhance academic reading performance and digital literacy engagement (George and Kumar, 2024; Fitriati and Williyan, 2025), the integration of CTS within technology-enhanced EFL instruction remains conceptually and pedagogically under-theorized. Most existing studies either evaluate CTS as a post-hoc learning outcome or describe artificial intelligence (AI) tools in terms of efficiency and engagement. However, they rarely specify how particular AI affordances may correspond to distinct cognitive dimensions of CTS in reading. As a result, there is limited guidance for instructors on how to deliberately design AI-supported activities that target specific forms of higher-order thinking.

This gap is particularly salient in assessment-driven educational systems such as China. In the Chinese EFL context, longstanding examination structures and teacher-centered instructional traditions often constrain opportunities for higher-order reasoning and dialogic inquiry (Du and Zhang, 2022; Yuan et al., 2022; Wang, 2024). While prior research has discussed cultural and institutional influences on classroom discourse practices (Coombe et al., 2020; Yin et al., 2023; Wolterinck et al., 2024), less attention has been paid to the role of AI-supported environments. These environments may either reinforce surface-level engagement or create new affordances for deeper cognitive processing. Consequently, a critical question arises: How can AI integration in blended reading contexts be intentionally designed to scaffold critical thinking, rather than merely automate lower-level tasks? Addressing this question requires identifying whether current uses of AI align with, under-support, or potentially bypass particular CTS dimensions.

Blended learning (BL), defined as the purposeful integration of face-to-face and online instruction, has been widely identified as a structure capable of supporting reflective and student-centered learning (Graham, 2006, 2013; Hrastinski, 2019; ElSayad, 2024). The addition of AI tools within BL environments introduces adaptive feedback mechanisms, real-time interaction, and personalized scaffolding. However, existing research has largely examined AI applications in terms of efficiency, engagement, or performance outcomes, with limited attention to how these functions may align with specific CTS dimensions in EFL reading (Yang et al., 2025). What remains insufficiently explored is the cognitive–technological alignment needed to translate AI affordances into meaningful CTS development. In particular, the field lacks an analytic framework that makes explicit which AI functions are pedagogically appropriate for supporting interpretation, analysis, evaluation, inference, explanation, or self-regulation in reading tasks.

To address this gap, the present study adopts an integrated theoretical lens that conceptualizes CTS development as a cognitive, pedagogical, and literacy-mediated process. First, Critical Thinking Skills Theory (Facione, 1990) provides a six-dimension cognitive structure (interpretation, analysis, evaluation, inference, explanation, and self-regulation) serving as a foundation for examining higher-order reasoning processes. Second, Blended Learning Theory (Graham, 2006) offers structural principles for understanding how instructional modalities can be orchestrated to support interaction and scaffolding. Third, Reading Theory (Grabe and Stoller, 2019) situates CTS within academic literacy practices, emphasizing strategic processing and metacognitive regulation in text comprehension. Rather than treating these perspectives as parallel frameworks, this study integrates them to examine how cognitive dimensions of CTS can be potentially supported through pedagogical design and technologically mediated reading processes.

Guided by McKillip’s (1987) Discrepancy Model, this study conducts a discrepancy-based needs analysis to examine learners’ current experiences, desired learning outcomes, and perceived gaps in AI-supported blended reading contexts. Unlike conventional needs analyses that primarily document learner deficiencies, this study conceptualizes discrepancy as a structural misalignment. This misalignment occurs between (a) targeted CTS dimensions and (b) the ways AI tools are currently used in blended reading tasks. By identifying patterns of misalignment and unmet cognitive needs, the study examines the gaps between Chinese EFL undergraduates’ current and desired CTS practices in AI-supported blended reading contexts. It also generates empirical insights to inform the alignment of AI affordances with CTS dimensions in AI-supported blended reading (ABR) module design. Based on these findings, the study proposes a design-oriented Technology–CTS Needs Matrix that integrates empirical findings from participant data with literature- and theory-informed design extrapolations, specifying (1) which CTS dimensions are insufficiently supported, (2) which AI affordances are predominantly used for lower-level processing, and (3) how instructional tasks can be redesigned to better target higher-order thinking. This matrix does not merely describe AI use; it provides dimension-level design guidance that can be examined and refined in future instructional implementations.

Specifically, the study addresses the following research questions:

1. How do Chinese EFL undergraduates currently experience and practice CTS in reading contexts?
2. How do students engage with AI tools in blended reading contexts, and what perceived affordances and constraints shape their use?
3. What discrepancies exist between students’ current practices and desired CTS-oriented learning outcomes?
4. How do the identified discrepancies potentially reveal structural misalignments between AI mediation and the development of CTS in blended reading contexts?

This study makes two concrete contributions. Theoretically, it reframes discrepancy-based needs analysis as a method for diagnosing the alignment between cognitive objectives and AI-supported learning environments. Practically, it proposes a matrix-based design framework that enables instructors to map specific AI functions to CTS dimensions and justify blended reading module design in transparent and replicable ways. The following section reviews relevant literature on CTS, reading theory, BL, and AI integration to situate this investigation within ongoing scholarly conversations.

## 2 Literature review

### 2.1 Reading and critical thinking skills in EFL contexts

Reading comprehension and CTS are closely intertwined in EFL education. Reading is an active cognitive process integrating linguistic decoding with higher-order skills such as interpretation, analysis, inference, and evaluation (Facione, 1990; Anderson and Krathwohl, 2001; Grabe and Stoller, 2019). In EFL contexts, this process is further complicated by limited linguistic proficiency and heavy reliance on test-oriented reading practices, and EFL learners must also assess authorial intent, detect bias, and synthesize multiple perspectives (Reiber-Kuijpers et al., 2021; Altun, 2023; Weng, 2023). However, many learners struggle to move beyond surface-level comprehension due to exam-oriented instruction, teacher-dominated classroom discourse, and limited analytical reading practice (Du and Zhang, 2022; Ali et al., 2023; Li and Zhu, 2023; Liu and Ren, 2024). These constraints often result in a disconnect between reading comprehension tasks and explicit CTS instruction, particularly in higher education EFL settings (Al-Darwish and Al-Sehli, 2023).

AI-supported learning environments have shown potential to address these challenges by providing adaptive feedback, contextual prompts, and metacognitive guidance that enhance inference-making and evaluative reasoning (Crompton et al., 2024; List et al., 2024; Daud et al., 2025). Unlike conventional digital tools, AI systems can respond dynamically to learners’ reading behaviors and cognitive states, thereby offering scaffolded support aligned with CTS development. Evidence from Chinese EFL learners using AI-supported informal digital learning of English indicates increased motivation, enjoyment, and self-regulation, supporting critical reading development (Liu et al., 2024a, 2024b). Meta-analyses further confirm that AI-enhanced informal learning can improve both language proficiency and CTS-related constructs such as self-regulated learning and motivation (Guan et al., 2024). Despite these promising findings, existing studies typically examine CTS development or AI-supported learning in isolation, without specifying how particular AI affordances may correspond to distinct cognitive dimensions of CTS during reading processes. Consequently, the literature offers limited guidance on how AI-supported environments can be intentionally structured to support dimension-specific critical thinking practices in EFL reading.

### 2.2 Blended and AI-supported models in EFL reading

Blended learning has become a central model for innovation in language education (Hrastinski, 2019; Graham and Halverson, 2022). In EFL reading, BL integrates classroom interaction with digital platforms, providing individualized learning pathways, learner autonomy, and opportunities to extend comprehension beyond literal meaning (Liu et al., 2020; Wang et al., 2023; Alazemi, 2024). Moreover, well-designed blended environments support CTS by allowing learners to revisit materials, engage in reflective and collaborative feedback, and benefit from technological tools such as learning management systems, online annotation platforms, and digital reading journals (Reiber-Kuijpers et al., 2021; Bervell et al., 2021; Cui, 2023; Huang et al., 2023). However, empirical findings also indicate that BL does not automatically foster CTS, and its potential effectiveness depends heavily on teacher readiness, learners’ self regulation, and instructional design quality, factors that remain underexplored in Chinese higher education (Martín-García et al., 2019; Cao et al., 2023). In poorly designed BL contexts, online components may merely replicate traditional comprehension exercises instead of promoting critical inquiry.

Building on this, the integration of AI further enhances BL by offering adaptive systems, intelligent tutoring, and data-driven analytics that provide personalized feedback and metacognitive support aligned with CTS development (Crompton et al., 2024; Fitriati and Williyan, 2025; Mizza et al., 2025). AI-supported BL thus represents a shift from content delivery toward process-oriented reading support, which can enable learners to monitor, evaluate, and adjust their reading strategies (Teng et al., 2024). Overall, AI-supported BL models appear well positioned to support CTS through structured, reflective, and learner-centered reading experiences. However, the existing literature rarely specifies how AI functions within blended environments may correspond to particular CTS dimensions in reading tasks. As a result, the design of AI-supported blended reading modules often lacks an explicit cognitive rationale linking technological affordances to targeted critical thinking processes.

### 2.3 Discrepancy-based needs analysis for an ABR module

Needs analysis (NA) is a crucial component of instructional design that ensures curricula align with learners’ goals, contexts, and challenges (Basturkmen, 2024). While traditional NA focused primarily on linguistic deficiencies, contemporary approaches increasingly address cognitive, metacognitive, and affective needs that are essential for fostering CTS (Brown, 2016; Richards and Pun, 2023). Among these approaches, McKillip’s (1987) Discrepancy Model remains influential, categorizing needs into current status (what is), ideal status (what should be), and the discrepancy, defined as the gap between the two. This model is particularly suitable for CTS-oriented instruction, as critical thinking skills development inherently involves progressive movement from existing practices to higher-order cognitive engagement. Applied to EFL reading, this framework systematically identifies learners’ perceived CTS levels, desired outcomes, and the instructional or technological supports that can help bridge this gap.

Such analysis is particularly valuable in AI-supported blended environments, where adaptive feedback can enhance learning but learners may still struggle to sustain critical engagement (Wu, 2024; Fitriati and Williyan, 2025). Without a systematic NA, AI tools may be underutilized or misaligned with learners’ actual needs and expectations. Recent research therefore calls for NA frameworks that incorporate AI literacy, digital readiness, and self-efficacy to better address evolving learning demands (Guan et al., 2024; Daud et al., 2025). A discrepancy-based NA integrating these dimensions can more comprehensively capture learners’ expectations toward an ABR module and identify the cognitive, affective, and technological factors that may influence CTS development. Importantly, when applied to AI-supported reading environments, discrepancy analysis can also reveal potential misalignments between learners’ desired critical thinking practices and the ways AI tools are currently used in learning tasks. This perspective allows needs analysis to move beyond identifying learner deficits toward diagnosing structural gaps between cognitive goals and technological mediation.

### 2.4 Research trends and gaps in CTS-oriented ABR module

Internationally, empirical research integrating CTS into EFL reading has expanded, with studies reporting improved analytical and argumentative performance in flipped and blended settings when online preparation aligns with in-class critical discussion (Birova et al., 2023; Qi et al., 2024). However, findings across contexts remain inconsistent, and evidence from the Chinese context remains limited and fragmented (Liu and Ren, 2024). University learners have demonstrated low metacognitive awareness and difficulty transferring CTS strategies across texts (Yang and Gamble, 2023). Although BL is gaining traction in Chinese EFL instruction due to its flexibility and engagement benefits (Cheng, 2025; Cao and Phongsatha, 2025), most implementations prioritize efficiency and examination performance rather than CTS as an explicit instructional goal (Er et al., 2025). Existing needs analyses have informed critical reading and interactive module design (Alim et al., 2025; He and AlSaqqaf, 2025), yet NA has rarely been systematically embedded as a foundational step in the design of the CTS-oriented ABR module.

Emerging AI-supported BL research has demonstrated the potential of adaptive tools, learning analytics, and self-regulated learning platforms to enhance metacognitive awareness and sustain critical engagement (Crompton et al., 2024; Liu et al., 2024a; Fitriati and Williyan, 2025; Liu et al., 2025). However, despite these technological affordances, limited attention has been paid to how such features may be aligned with learners’ perceived needs or informed by empirically grounded design rationales. In particular, few studies have examined how discrepancy-based needs analysis can be used to diagnose the alignment between learners’ current CTS practices, their desired critical reading outcomes, and the pedagogical use of AI tools within blended environments. Without such alignment, AI-supported learning may risk reinforcing lower-level processing rather than fostering critical thinking skills. Therefore, this study addresses this gap by conducting a discrepancy-based needs analysis to examine Chinese EFL undergraduates’ current experiences, desired CTS-oriented learning outcomes, and perceived use of AI in blended reading contexts. By identifying patterns of misalignment, the study aims to generate insights that can inform the design of a CTS-oriented ABR module. These insights are subsequently integrated into a design-oriented Technology–CTS Needs Matrix, which combines empirical findings with literature- and theory-informed design extrapolations to guide AI-supported instructional design.

## 3 Methodology

### 3.1 Research design

This study employed a qualitative multi-phase design grounded in discrepancy-based needs analysis to examine the needs for developing CTS within an ABR module for Chinese EFL undergraduates. The research followed a sequential two-stage structure. Stage one involved expert review of the semi-structured interview guide and the conceptual design of the AI-assisted reading prototype to establish content validity (Lynn, 1986) and ensure alignment with the study’s conceptual framework. Stage two focused on empirical data collection using four complementary tools: the semi-structured interview guide, the AI-assisted reading prototype, reflective learning logs, and a literature–technology scan. The semi-structured interviews and reflective learning logs constituted the primary empirical sources from which the study’s themes were generated regarding learners’ CTS needs in AI-supported blended reading, while the AI prototype and literature–technology scan provided supplementary descriptive and contextual evidence. In combination, these tools identified learners’ current practices, desired outcomes, perceived gaps, and design implications for the module. This structure strengthened instrument validity and enhanced the credibility of findings through rich, contextualized learner perspectives, aligning with calls for context-sensitive, empirically grounded needs analyses in language education (Brown, 2016; Park, 2022) and for technology-informed curriculum design that improves pedagogical relevance (Ke and AlSaqqaf, 2024).

### 3.2 Participants and setting

A panel of nine experts in English language education, critical thinking pedagogy, and educational technology validated the semi-structured interview guide and AI-assisted reading prototype. They were purposively selected based on their academic qualifications and research expertise. The panel included four professors, three associate professors, and two senior lecturers, all with over 10 years of higher education teaching and research experience. Semi-structured interviews were conducted with 20 Chinese EFL undergraduates from Z University (a pseudonym) in eastern China. Prior to the interviews, participants engaged with the AI-assisted reading prototype and then completed reflective learning logs, which enabled them to record their experiences and perceptions regarding the prototype. Purposive sampling was employed to ensure diversity in academic majors, English proficiency levels, and prior exposure to AI-supported or BL platforms. All participants had completed at least one EFL reading course and had prior exposure to digital learning platforms (e.g., Zoom, Rain Classroom) that occasionally featured interactive tools, ensuring they could engage meaningfully with the AI-assisted reading prototype.

### 3.3 Data collection tools

Multiple data collection tools were employed to explore Chinese EFL undergraduates’ needs for developing CTS in an AI-supported blended reading context. The primary instrument was a semi-structured interview guide, supplemented by an AI-assisted reading prototype, reflective learning logs, and a literature–technology scan. Each tool served a distinct role in triangulating data and enhancing the depth and credibility of the needs analysis.

#### 3.3.1 AI-assisted reading prototype

To elicit concrete learner reflections on AI-supported reading, an exploratory prototype was implemented using the ChatGPT API provided by OpenAI (GPT-4o model) (see Appendix A). The prototype provided a uniform hands-on experience and functioned solely as a data elicitation stimulus rather than an instructional module. Its outputs were used only descriptively to provide supplementary interpretive support and were not used as a basis for theme generation. It illustrated three potential AI affordances relevant to CTS-oriented reading: (1) adaptive text simplification aligned with learners’ proficiency levels; (2) contextual prompts targeting key CTS such as interpretation, analysis, evaluation and inference; and (3) automated feedback on reflective responses.

The prototype was configured with standardized system instructions positioning the AI as a reading facilitator that supported learners’ critical engagement with texts rather than providing direct answers. To ensure consistency across participants, a fixed sequence of learner prompts was implemented: (1) Interpretation task: “What is the main idea of the passage, and how do you interpret the author’s position?”; (2) Analysis task: “How does the author support the main argument in the passage?”; (3) Evaluation task: “How convincing are the arguments presented in the passage? Provide textual evidence to support your evaluation.”; and (4) Inference task: “What can you infer from this passage about the author’s perspective or assumptions?” The system instructions further guided the AI to assist learners by providing simplified explanations of passages when clarification was requested or when responses indicated misunderstanding. The AI also generated reflective prompts aligned with CTS dimensions and offered brief formative feedback focusing on the clarity of the learner’s reasoning and the use of textual evidence to justify claims. To maintain procedural consistency across participants, the model temperature was set at 0.3 to reduce output variability while preserving natural language interaction.

Adaptive text simplification and feedback on reasoning quality were implemented through explicit operational rules embedded in the system prompt to ensure consistent AI behavior across interactions. For adaptive simplification, the AI first evaluated sentence length and syntactic complexity and applied simplification when passages were likely to create comprehension difficulty for learners. The process involved lexical substitution rules that replaced low-frequency vocabulary with more common equivalents and the restructuring of complex sentences into shorter and more accessible forms. Non-essential subordinate clauses were removed when they did not affect the core informational meaning of the passage. These procedures were designed to preserve the original semantic content while reducing linguistic complexity and cognitive load.

Feedback on learners’ reasoning quality followed three explicit criteria derived from Facione’s (1990) CTS framework: (a) relevance of textual evidence, referring to whether responses cited specific passages or ideas from the text; (b) clarity of explanation, referring to whether reasoning was expressed in a coherent and understandable manner; and (c) logical justification, referring to whether claims were supported by defensible arguments. The AI evaluated learner responses against these criteria and generated brief formative feedback highlighting strengths or suggesting ways to improve reasoning. For example, feedback could state: “Your explanation is clear, but consider citing evidence from paragraph 3 to strengthen your argument.”

The standardized prompt sequence, operational logic for simplification, and explicit feedback criteria ensured that all participants interacted with the prototype under comparable conditions. This design allowed variations in responses to reflect learners’ reasoning processes rather than differences in system input, thereby supporting methodological transparency and enabling replication in future studies.

#### 3.3.2 Reflective learning logs

Reflective learning logs were employed to capture participants’ self-regulated engagement with digital and AI tools during reading. Data were collected via Wenjuanxing, a widely used online survey platform in Chinese educational research, enabling participants to submit open-ended reflections. Each log was systematically structured around three prompts addressing learners’ perceived challenges when using AI in blended reading, the influence of AI feedback on the development of their critical thinking skills, and the strategies they adopted to balance AI support with independent thinking. The logs generated qualitative data that complemented interview findings and provided deeper insights into learners’ engagement across both AI-supported and traditional reading contexts. These logs served as a primary data source for thematic analysis and theme generation.

#### 3.3.3 Semi-structured interview guide

The interview guide was developed based on McKillip’s (1987) Discrepancy Model, which conceptualizes learners’ needs across three dimensions. Question design also drew upon Facione’s (1990) six core skills to ensure comprehensive coverage of reading-related critical thinking skills. The guide comprised 13 open-ended questions organized into four sections (see Appendix B), exploring learners’ reading experiences, expectations for improvement, and perceived gaps in instructional support, BL environments, and AI-assisted scaffolding. Interview data also served as a primary data source for thematic analysis and theme generation.

#### 3.3.4 Literature–technology scan

To contextualize the study and guide interpretation of empirical findings, a targeted literature–technology scan was conducted as a secondary analytic procedure. Ten studies published between 2020 and 2025 (see Appendix C) on AI-supported and technology-enhanced reading platforms within BL contexts were reviewed, focusing on technological affordances, pedagogical orientations, and integration practices. This literature–technology scan informed the interpretation of empirical findings and guided design extrapolations, but it did not itself generate the thematic categories derived directly from participant data.

### 3.4 Research procedure

The study adopted a multi-phase, multi-tool design to ensure methodological rigor, instrument validity, and triangulated insights into learners’ needs for CTS development in ABR module. The procedure was implemented in six sequential phases: preparatory expert validation, exploratory AI-mediated interaction, reflection and instrument refinement, student interviews, literature–technology scan, and data organization and analysis. Only the semi-structured interviews and reflective logs were used for final thematic coding, while outputs from the AI-assisted reading prototype and the literature–technology scan served as supporting descriptive sources.

#### 3.4.1 Preparatory expert validation

The prototype and the interview guide were evaluated by a panel of nine experts for relevance, clarity, simplicity, CTS coverage, and alignment with AI-supported BL contexts. Feedback was synthesized to refine the interview guide, strengthen the coherence between McKillip’s (1987) discrepancy dimensions and Facione’s (1990) CTS framework, and confirm the prototype’s feasibility. Following revisions, three experts reassessed the materials to verify their suitability for the study context, ensuring content validity and pedagogical relevance.

#### 3.4.2 Phase 1: exploratory AI-mediated interaction

Participants explored the AI-supported blended reading approach by interacting with the AI-assisted reading prototype, which provided feedback on their responses. Twenty undergraduates from Z University engaged in an exploratory activity using a standardized reading excerpt, “The Role of Curiosity in Learning”, accompanied by a standardized sequence of reasoning prompts targeting four CTS dimensions (interpretation, analysis, evaluation, and inference), implemented through the four-task prompt structure described in Section 3.3. Initial prompts were identical for all participants to ensure consistency, while the system generated adaptive feedback, clarification questions, and reflection triggers based on individual responses. Sessions lasted 20–30 min, with participants submitting a one-page screenshot report (see Appendix D) capturing their responses and the AI-generated feedback for qualitative analysis and cross-phase triangulation. This phase familiarized participants with the AI-supported reading environment and provided a foundation for subsequent reflective logging and interviews.

#### 3.4.3 Phase 2: reflection and instrument refinement

After completing the AI-supported reading task, participants submitted reflective learning logs via Wenjuanxing, capturing cognitive, affective, and technological aspects of their experiences and CTS needs. The logs served as a key data source and informed refinement of the interview guide. Two experts subsequently re-evaluated the revised guide to ensure conceptual clarity, contextual relevance, and suitability for the next phase of data collection.

#### 3.4.4 Phase 3: student interviews

This phase constituted the main qualitative inquiry of the study. Building on prior phases, individual semi-structured interviews were conducted using the validated guide, refined based on reflective learning logs. Twenty interviews were held online via Tencent Meeting, each lasting 25–35 min and audio-recorded with participant consent. Interviews were primarily conducted in Chinese, with occasional English for reading or AI terminology. Researchers acted as neutral facilitators, encouraging elaboration while maintaining procedural consistency. All interview data were anonymized, translated into English, and reviewed by bilingual research assistants for linguistic accuracy and clarity.

#### 3.4.5 Phase 4: literature–technology scan

To contextualize findings within current pedagogical and technological practices, a targeted literature–technology scan was conducted. Ten studies published between 2020 and 2025 were purposively selected from Scopus and Web of Science, covering four dimensions: reading, CTS, BL, and AI-supported or technology-enhanced pedagogical approaches. This phase complemented the empirical data from Phases 1–3 and informed design extrapolations, providing literature- and theory-informed guidance for instructional recommendations, rather than producing additional empirical categories.

#### 3.4.6 Final phase: data organization and analysis

**Figure 1:**
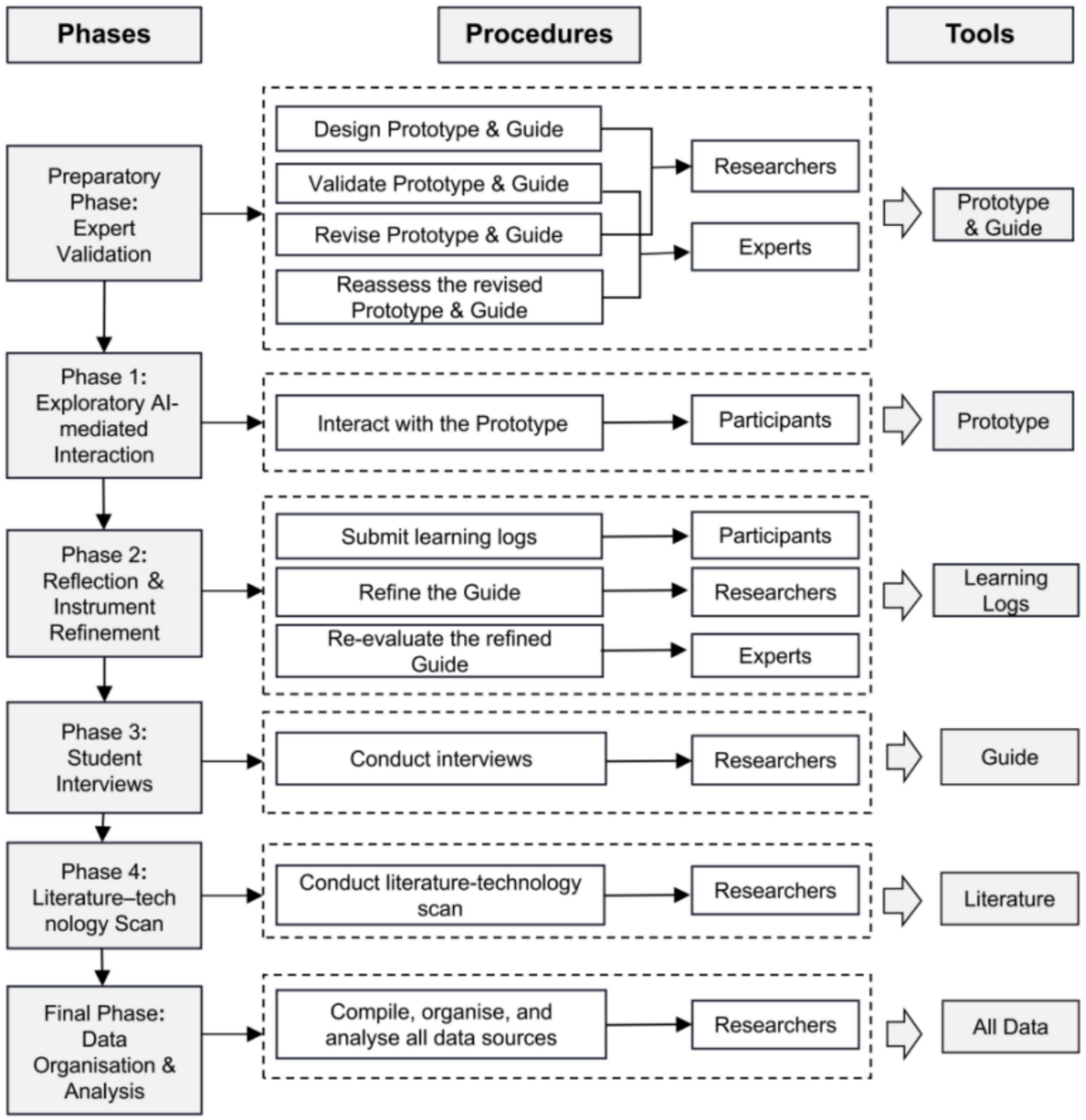
Research procedures of the study.

All data sources, including expert validation, AI-mediated reading interaction records, reflective learning logs, interview data, and findings from the literature and technology scan, were systematically compiled and organized. Manual thematic analysis of interviews and reflective learning logs formed the basis of the final emergent themes, whereas AI-mediated outputs and findings from the literature–technology scan served as supplementary descriptive and contextual evidence. NVivo 14 was used to support the management, coding, and thematic analysis of qualitative data through triangulation across phases and data types. This approach strengthened cross-source verification of emerging categories and supported a comprehensive understanding of learners’ experiences, needs, and perceptions of CTS within the AI-supported BL context. An overview of the research procedures is presented in Figure 1.

### 3.5 Data analysis

All qualitative data were systematically organized and analyzed to align with the multi-phase design and enable triangulated interpretation. The dataset included AI-mediated reading interaction reports, reflective learning logs, interview transcripts, and findings from the literature–technology scan. NVivo 14 was used to facilitate data management and coding. A hybrid thematic analysis was employed, combining manual coding with AI-assisted support to strengthen analytic rigor and cross-source validation, without inferring causal effects or effectiveness of the AI-assisted intervention.

#### 3.5.1 Manual thematic analysis

Manual coding followed Braun and Clarke’s (2006) six-phase thematic analysis, operationalized in Forbes (2021), including data familiarization, initial coding, theme development and review, theme definition, and analytic reporting. Deductive codes were derived from Facione’s (1990) six core critical thinking skills and McKillip’s (1987) three needs dimensions to establish an initial coding framework, while inductive codes captured emergent themes related to learners’ experiences with the AI-supported BL approach, cognitive strategies, and emotional engagement. Each CTS dimension was operationalized based on the cognitive processes reflected in learner statements. For instance, statements involving identifying main ideas or textual meaning were coded under interpretation, those describing reasoning about relationships between ideas were coded as analysis, while expressions related to judging argument quality or credibility were categorized as evaluation. Difficulties in monitoring comprehension or reflecting on reading strategies were coded under self-regulation. This manual analysis formed the basis for generating the final themes. NVivo 14 facilitated systematic code organization and cross-source comparison across interview and reflective log datasets. To enhance trustworthiness, 20% of the data were independently double-coded by a second researcher, yielding a high level of inter-coder agreement (Cohen’s *κ* = 0.86). Analytic credibility was further strengthened through member checking and peer debriefing to reduce researcher bias and improve transparency.

To operationalize McKillip’s (1987) discrepancy-based needs analysis, qualitative data were coded according to three analytical categories: *current state*, *desired state*, and *discrepancy*. A concise set of coding rules with operational definitions and indicators was developed to ensure consistent interpretation of participant statements across interview transcripts and reflective learning logs. *Current state* referred to learners’ descriptions of their existing reading practices, difficulties, or experiences in AI-supported or blended learning contexts. *Desired state* captured participants’ expectations, preferred learning conditions, or perceived forms of support for developing critical thinking skills. *Discrepancy* referred to the gap between these two conditions, indicating areas where learners perceived their present learning experiences as insufficient to achieve their desired learning outcomes.

When participants expressed both problems and expectations within the same excerpt, statements were segmented analytically and coded separately according to their functional meaning. For example, the interview excerpt “I usually just read for the main idea, but I wish there were more questions that helped me analyze the author’s argument” was coded as follows: “I usually just read for the main idea” was categorized as *current state*, whereas “I wish there were more questions that helped me analyze the author’s argument” was coded as *desired state*. The contrast between these segments was interpreted as a *discrepancy*, indicating that current reading practices provided limited opportunities for analytical engagement. This coding logic enabled systematic identification of learners’ needs by mapping tensions between existing practices and desired learning conditions.

#### 3.5.2 AI-assisted NLP analysis

To complement manual thematic analysis and enhance analytic transparency, AI-assisted natural language processing (NLP) techniques were employed as supplementary descriptive analyses. The primary purpose of these techniques was to provide an additional computational perspective on the qualitative dataset and to examine whether machine-identified semantic patterns broadly converged with the themes derived from human coding. Specifically, BERTopic topic modeling was applied to explore latent semantic structures across interview transcripts and reflective learning logs. The BERTopic model was implemented using the sentence-transformer embedding model all-MiniLM-L6-v2. The minimum topic size was set to 10, and all other parameters were left at their default settings. This procedure enabled the identification of clusters of semantically related responses within the dataset. Topic interpretability was assessed through manual inspection of the most representative keywords and documents associated with each topic. The resulting topic clusters were subsequently compared with manually derived codes to examine conceptual overlap and interpretive consistency with the themes identified through manual thematic analysis. In addition, sentiment analysis was conducted using the TextBlob Python library to generate a descriptive overview of participants’ affective orientations toward the AI-supported blended reading (ABR) module. This analysis was intended to provide a general descriptive indication of emotional polarity across the dataset rather than to perform inferential or predictive analysis.

Prior to analysis, textual data were anonymized and preprocessed through tokenization, stop-word removal, and lemmatization to ensure consistency and reliability. The final themes reported in this study were generated solely through manual thematic analysis of interview transcripts and reflective learning logs. The NLP outputs provided descriptive triangulation and computational insights, and did not contribute to the development or labeling of themes. An example of interview extracts, including deductive and inductive codes as well as corresponding NLP outputs, is presented in Appendix E.

#### 3.5.3 Integration of learning logs and interview data

Reflective learning logs and interview transcripts were systematically integrated to support triangulated analysis across cognitive, affective, and technological dimensions. The logs captured immediate, context-specific reflections on AI-mediated reading, while interviews provided deeper narrative elaborations of learners’ needs. This integration served three purposes: (1) cross-validation of findings, enhancing credibility and reducing single-source bias; (2) comprehensive understanding, capturing convergent and divergent patterns in learners’ CTS development and engagement with the AI-supported blended reading approach; (3) foundation for subsequent analysis, informing the construction of the Technology–CTS Needs Matrix linking CTS dimensions to AI and digital affordances. These data served as the primary sources for theme generation.

#### 3.5.4 Mapping CTS to technological needs

The final analytic stage integrated coded data into a Technology–CTS Needs Matrix, presented explicitly as a design-oriented synthesis rather than a purely empirical framework, linking learners’ CTS dimensions with corresponding AI and digital affordances identified in the study and in the literature–technology scan. This design-oriented matrix combines participant-derived needs with evidence from prior studies to provide a theory-informed guide for aligning CTS development with AI-supported blended reading strategies, and is explicitly intended as a guiding framework for instructional design rather than being fully established by empirical data alone. Themes for the matrix were generated from interview transcripts and reflective learning logs, while AI outputs and the literature–technology scan served only to provide contextualization and cross-validation.

#### 3.5.5 Trustworthiness, reliability, and validity

To ensure methodological rigor, this study adhered to the qualitative research criteria of credibility, dependability, confirmability, and transferability (Lincoln and Guba, 1985; Erlandson et al., 1993). Multiple strategies were employed to enhance the accuracy, consistency, and transparency of the findings. Credibility was established through expert validation of the interview guide and AI-assisted reading prototype, ensuring alignment with CTS dimensions and AI-supported BL contexts. Member checking with three participants verified that emergent themes authentically reflected learners’ experiences (Creswell and Poth, 2018). Dependability was reinforced by independent double-coding of a subset of interview transcripts, with inter-coder reliability confirmed through iterative discussion and consensus-building (Miles et al., 2014). Detailed documentation of coding decisions and analytic memos created an explicit audit trail, supporting transparency and replicability. Triangulation across interviews, reflective logs, AI interaction records, and supporting literature further reduced bias and strengthened interpretive validity (Patton, 2015). Confirmability was achieved through comprehensive documentation of analytic procedures, while transferability was enhanced via rich, contextualized descriptions of participants and learning environments, allowing readers to assess applicability to similar educational contexts (Nowell et al., 2017). Collectively, these strategies ensured that the study’s interpretations were credible, dependable, and methodologically robust.

#### 3.5.6 Ethical considerations

Ethical approval for this study was obtained from the Science and Education Department Ethics Committee of the Second Affiliated Hospital of Xiamen Medical College (Approval No.: 20251210). Written informed consent was obtained from all participants prior to participation. Participants were informed of the voluntary nature of the study and their right to withdraw at any time (American Psychological Association, 2017; British Educational Research Association, 2018). To ensure confidentiality, pseudonyms were used and all digital data were stored on a password-protected external drive accessible only to the researchers.

## 4 Results

### 4.1 Overview of the results

Data were drawn from 20 AI-mediated interaction records, 20 reflective learning logs, 20 semi-structured interviews, and AI-assisted NLP outputs. Analysis of the final themes, derived from interviews and reflective logs, revealed patterned discrepancies between learners’ reported reading practices and their aspirations for critical engagement in AI-supported BL contexts. Three major empirical domains emerged: (1) Predominantly surface-level reading practices; (2) Aspirations for structured cognitive scaffolding; (3) Systemic gaps between AI availability and cognitive utilization. AI-assisted NLP analysis largely converged with manually derived themes, suggesting the descriptive validity of identified patterns but not contributing to theme generation.

### 4.2 Current state

#### 4.2.1 Predominance of literal processing

Eighteen of the 20 participants described their English reading as primarily comprehension-oriented, focusing on vocabulary, main ideas, and test-related questions. Only two reported regularly engaging in independent evaluation of arguments. Participants repeatedly framed reading as “understanding content” rather than interrogating reasoning: *“We seldom analyze why the author thinks that way; we just try to understand the content and answer the comprehension questions”* (P1). *“Reading is mostly about getting the main idea or understanding the author’s opinion”* (P2). Even during the AI-mediated task, responses tended to summarize rather than critique. Few participants extended answers beyond prompt requirements unless explicitly guided. However, limited variation was observed. Two higher-proficiency students described occasionally questioning authorial stance independently, though without systematic strategy use. This pattern suggests a descriptive orientation toward textual comprehension rather than implying causal limitations in analytical engagement.

#### 4.2.2 Weak metacognitive monitoring

Fourteen participants acknowledged rarely reviewing or revising their reasoning during reading. Reflective logs showed minimal explicit reference to strategy adjustment, reasoning checks, or self-correction. As one participant stated: *“I seldom look back to check why I misunderstand a passage”* (P3). Another explained: *“I know I should monitor my understanding, but I do not really know how to do it”* (P4). Only three participants described consistent reflective practices such as note-taking or reasoning tracking. Across data sources, metacognitive engagement appeared episodic rather than systematic.

#### 4.2.3 Instrumental AI use

All participants reported prior use of AI tools (e.g., ChatGPT, Grammarly, Kimi, Deepseek, Doubao). However, 16 out of 20 described using AI primarily for translation, grammar checking, summarization, or outline generation. As one participant stated: *“I often use ChatGPT to check grammar or make an outline”* (P5). Another participant stated: *“AI helps me summarize, but it does not teach me how to think”* (P6). Only four participants reported attempting to use AI for argument comparison or evaluative discussion, and even these attempts were described as unsystematic. Participants frequently expressed uncertainty about how to formulate prompts that stimulate deeper reasoning.

### 4.3 Desired state

While current practices were largely surface-level, participants articulated consistent aspirations for deeper cognitive engagement. The emergent themes reflecting these aspirations were generated from interviews and reflective logs. Three recurring patterns emerged: (1) explicit scaffolding of CTS, (2) interactive and collaborative learning support, and (3) AI-assisted adaptive feedback and personalization. Collectively, these dimensions represent learners’ articulated expectations and can inform the design of ideal environments for fostering CTS in blended reading contexts.

#### 4.3.1 Explicit scaffolding of CTS

Fifteen participants emphasized the need for structured guidance in applying CTS during reading. Several expressed a desire for explicit modelling and procedural support. As one participant noted, “*If the teacher can show examples of how to analyze an argument or evaluate evidence, I can follow that logic in my own reading”* (P7). Another similarly requested *“step-by-step guidance on questioning the author and checking evidence”* (P8). Visual scaffolds were also mentioned as facilitating cognitive transparency, with one student observing that *“argument maps make it easier to see how to think critically”* (P9). These accounts suggest a consistent demand for explicit and structured cognitive scaffolding to support the development of critical thinking skills in reading practices.

#### 4.3.2 Interactive and collaborative learning support

Thirteen participants emphasized the role of dialog and peer interaction in fostering CTS. Many described discussion as a mechanism for exposing alternative interpretations and prompting self-questioning. As one participant noted, *“Usually talking about the reading with classmates helps me notice different interpretations and question my own”* (P10). Similarly, another remarked that *“peer interaction makes me reflect on my own thinking and consider alternative perspectives”* (P11). Several participants expressed a preference for blended formats that combine face-to-face discussion with online collaborative tools. One explained, *“I like combining classroom discussion with online forums; it helps me explain my reasoning and see others’ ideas”* (P12), highlighting the perceived value of multimodal dialog for articulating and negotiating meaning. Participants also underscored the importance of teacher facilitation that supports reasoning without prematurely closing inquiry. As one student stated, *“Teachers should not give the answer too early, but guide us to think step by step”* (P13). Across participants, dialog appears to function as a central mechanism for stimulating reflective and evaluative engagement with texts.

#### 4.3.3 AI-assisted adaptive feedback and personalization

Participants envisioned AI tools that monitor reading behavior, identify reasoning gaps, and provide personalized support for CTS development. Eleven participants articulated a preference for AI functioning as a “thinking partner” rather than an answer provider, capable of prompting deeper reflection through context-sensitive questioning. As one participant explained, *“It’d be great if the AI could let me compare different viewpoints. If the AI can ask why I agree or disagree with the author, I will think more carefully”* (P14), underscoring the perceived value of dialogic prompting. Fourteen participants emphasized the importance of feedback focused on reasoning quality rather than correctness alone. One noted, *“I want AI to give me feedback on how good my reasoning is, not just the correct answer”* (P15), while another suggested that *“dashboards showing my progress in thinking skills would help me track improvement”* (P16), highlighting expectations for visible and developmental assessment mechanisms. Motivational affordances were also mentioned, with one participant observing that *“interactive or gamified AI tasks would make reading more engaging”* (P17). At the same time, participants consistently positioned AI as supplementary to human instruction. As one stated, *“AI can guide us, but teachers help us truly understand”* (P18), indicating a perceived boundary between technological scaffolding and pedagogical authority. Across accounts, AI was conceptualized by participants as a reflective and adaptive scaffold that could potentially support teacher-supported critical engagement.

### 4.4 Discrepancies between current and desired conditions

Cross-analysis of interview transcripts, reflective logs, and AI-mediated interaction records revealed systematic discrepancies between participants’ reported practices and their articulated expectations for developing critical thinking skills in AI-supported blended reading contexts. These discrepancies emerged not as isolated inconsistencies but as recurring structural patterns across datasets. Participants consistently endorsed the importance of critical engagement, yet many expressed uncertainty regarding acceptable standards of reasoning in academic reading. While critical thinking was rhetorically emphasized, procedural indicators of how such engagement should be enacted remained under-specified. Descriptions of classroom and online reading activities frequently centered on vocabulary acquisition, comprehension questions, and submission-oriented platform use, indicating that endorsement of critical thinking coexisted with limited structured reasoning practice.

Although regular access to AI tools was universal, usage patterns were predominantly instrumental. Translation, grammar checking, summarization, and outline generation were referenced more frequently than evaluative comparison, argument interrogation, or dialogic reasoning. Many participants reported difficulty determining the reliability or contextual appropriateness of AI-generated responses and indicated the need for teacher guidance when attempting deeper engagement. Similarly, peer discussion was often framed as a mechanism for checking understanding rather than advancing analytical depth, and explicit descriptions of independently implemented reasoning strategies were limited across data sources. This pattern suggests reliance on externally provided scaffolding without consistent internalization of strategic reasoning practices.

The findings suggest aligned structural tensions: endorsement of critical thinking skills alongside limited procedural clarity; AI accessibility alongside constrained evaluative utilization; and articulated need for scaffolding alongside limited strategy awareness. Convergence across qualitative data sources and AI-assisted NLP outputs supports the consistency of these observed patterns, pointing to systemic misalignment between pedagogical design, technological mediation, and learners’ metacognitive development.

### 4.5 Synthesis of findings across research questions and CTS dimensions

The findings can be synthesized in relation to the four research questions and the six CTS dimensions proposed by Facione (1990). RQ1 examined how learners currently experience and practice CTS during English reading. Evidence from interview data, reflective learning logs, and AI-mediated interaction records indicates that students’ reading practices are predominantly oriented toward interpretation and basic explanation, focusing on vocabulary comprehension and identification of main ideas. Higher-order CTS dimensions, particularly analysis, evaluation, inference, and self-regulation, were rarely enacted in participants’ descriptions of their reading practices, indicating limited engagement with structured reasoning processes.

RQ2 explored how learners engage with AI tools within blended reading contexts. The findings show that AI is primarily used for instrumental linguistic support, including translation, grammar checking, summarization, and outline generation. These practices mainly facilitate lower-level interpretation and language processing rather than higher-order reasoning. Only a small number of participants reported using AI to compare viewpoints or question arguments, indicating limited engagement with the CTS dimensions of analysis, evaluation, and inference.

RQ3 focused on the discrepancies between learners’ current practices and their desired learning conditions. The analysis revealed systematic gaps across three dimensions: (a) endorsement of critical thinking alongside limited procedural understanding of how to enact it; (b) widespread access to AI tools alongside predominantly instrumental use; and (c) learners’ expressed need for cognitive scaffolding alongside limited strategy awareness. These discrepancies suggest a structural misalignment between learners’ aspirations for deeper reasoning and the current instructional and technological practices shaping their reading experiences.

RQ4 addresses how these discrepancies reveal broader structural misalignments between AI mediation and CTS development. Although AI tools are widely available, their pedagogical use is not systematically aligned with the six CTS dimensions. Learners emphasized the potential value of AI tools that provide adaptive prompts, dialogic questioning, and feedback on reasoning quality, particularly when integrated with teacher guidance and peer discussion. Thus, these findings informed the preliminary development of the Technology–CTS Needs Matrix, framed as a design-oriented synthesis that integrates participant-derived needs with AI-supported instructional affordances identified in the literature, rather than as a framework fully established by empirical data, linking specific CTS dimensions with corresponding AI and pedagogical affordances in AI-supported blended reading environments.

## 5 Discussion

The findings suggest a structural imbalance between linguistic skill acquisition and higher-order cognitive engagement in AI-supported blended reading contexts. When interpreted through Facione’s (1990) six-dimensional framework of CTS, learners’ reported practices primarily reflect engagement at the levels of interpretation and surface-level explanation. In contrast, analysis, evaluation, inference, and particularly self-regulation appear comparatively underdeveloped. Eighteen participants described reading as focusing on vocabulary, main ideas, and comprehension tasks, while only two reported engaging in independent evaluation of arguments. This uneven distribution across cognitive dimensions indicates that CTS are acknowledged in principle but are not systematically embedded as a procedural component of reading instruction.

The limited presence of self-regulation is especially significant. Fourteen participants acknowledged rarely reviewing or revising their reasoning, and reflective logs contained minimal reference to strategy monitoring or adjustment. Within the CTS framework, self-regulation functions as the coordinating dimension that enables learners to examine the quality of their interpretation, analysis, and evaluation. However, its presence in the data appears episodic and unsystematic. This pattern suggests that higher-order reasoning has not been consistently internalized. Learners’ statements that they “know” they should monitor comprehension but lack clarity about how to do so further illustrate structural misalignment between AI affordances, instructional design, and critical thinking development.

Reading Theory (Grabe and Stoller, 2019) situates critical thinking within academic literacy practices that integrate strategic processing and metacognitive regulation. The predominance of comprehension-oriented descriptions and task completion suggests that reading remains focused on content understanding rather than systematic interrogation of argument structure or evidential support. Moreover, there is limited evidence of strategic language use across interviews and reflective logs. This suggests that analytical and evaluative processing has not been proceduralized within routine reading activities. In this sense, the findings point to restricted alignment between cognitive strategy instruction and AI-mediated reading affordances.

Blended Learning Theory (Graham, 2006) provides insight into the structural conditions shaping these patterns. Although AI tools are universally accessible among participants, their reported use is predominantly instrumental. Sixteen participants described translation, grammar checking, or summarization as primary applications, while only three reported attempting evaluative comparison or argument discussion. The blended environment therefore appears to provide technological access without consistent cognitive orchestration. Without explicit modelling, guided questioning, and structured interaction cycles, AI tools may tend to support efficiency in task completion rather than structured reasoning practice. These findings suggest that modality integration alone does not ensure cognitive depth. Pedagogical and technological alignment remains decisive.

Guided by McKillip’s (1987) Discrepancy Model, three interrelated gaps can be identified. First, there is a discrepancy between learners’ endorsement of critical thinking skills and the limited procedural clarity regarding how it should be enacted in reading tasks. Second, a gap exists between AI availability and evaluative utilization, as access to technological tools does not translate into systematic reasoning support. Third, learners express a strong need for teacher modelling, dialogic interaction, and adaptive feedback. However, they demonstrate limited internalized strategy use across data sources. These discrepancies are concentrated primarily in the dimensions of analysis, evaluation, inference, and self-regulation rather than in basic comprehension.

Importantly, the findings do not indicate motivational resistance. Participants consistently expressed aspirations for deeper cognitive engagement and articulated coherent expectations for structured scaffolding. The misalignment therefore appears structural rather than dispositional. It may reflect insufficient integration among cognitive objectives, instructional design, and AI mediation within the BL ecology. Therefore, the results suggest that effective AI-supported blended reading may require deliberate alignment among AI affordances, instructional design, and the six CTS dimensions, explicit metacognitive strategy training, and structured orchestration of technological support. Reading tasks may need to be designed in ways that make analytical questioning, evidential evaluation, and reflective monitoring procedurally visible. AI may serve as a guided scaffold that prompts reasoning and supports feedback on cognitive quality rather than serving solely as a linguistic assistance tool.

As a result, these structurally identified discrepancies indicate the need for a systematic design response rather than isolated instructional adjustments. To address these discrepancies in a systematic manner, a design-oriented Technology–CTS Needs Matrix was constructed, explicitly distinguishing between empirical findings and design extrapolations. The matrix integrates participant-derived needs (empirical findings) with AI-mediated instructional affordances identified from the literature and pedagogical reasoning (design extrapolations). It foregrounds misalignment as a central analytical lens to inform design decisions. It is presented as a guiding conceptual synthesis rather than a framework fully validated by empirical data. The following Technology–CTS Needs Matrix (Table 1) is presented as a structured guide to inform instructional design in AI-supported blended reading. It integrates empirically identified learner needs with AI-supported instructional affordances reported in the literature, while maintaining a design-oriented perspective. It is intended to guide the design of AI-supported blended reading interventions as a set of guiding principles, rather than to serve as a fully empirically validated framework. It does not provide evidence of instructional effectiveness, but instead offers a structured basis for future design and empirical testing. Specifically, the matrix can be used by instructors to identify underdeveloped CTS dimensions in reading tasks, select corresponding AI-supported strategies, and design activities that more explicitly target higher-order cognitive processes.

**Table 1:** Technology–CTS needs matrix.

CTS	Identified learners’ needs	Potential AI/digital support	Supporting sources
Interpretation	Difficulty identifying key ideas, textual relationships, and implicit meanings in academic readings.	AI-based scaffolding tools (contextual clarification, adaptive text simplification, multimodal reading aids).	Murphy Odo (2023), Crompton et al. (2024), and Bahrainian et al. (2024)
Analysis	Limited ability to deconstruct arguments and link evidence to claims.	Digital annotation platforms and interactive reasoning maps for collaborative textual analysis.	Chen et al. (2020) and Du et al. (2022)
Evaluation	Need for guided practice in assessing source credibility and argument soundness.	AI-generated evaluative prompts and automated feedback on reasoning quality.	Fitriati and Williyan (2025), Weidlich et al. (2025), and Liu et al. (2025)
Inference	Insufficient strategies for drawing logical conclusions and identifying implied relationships.	Adaptive reading tasks with AI-driven question generation and inference feedback.	Steuer et al. (2022) and Crompton et al. (2024)
Explanation	Challenges in articulating reasoning processes and synthesizing multiple viewpoints.	Collaborative peer discussion boards and AI-supported reflective writing prompts.	Zhu et al. (2020), Oyarzun and Martin (2023), and Tian and Zheng (2024)
Self-regulation	Limited metacognitive monitoring and reflection on reading-thinking processes.	Learning logs, AI-based feedback dashboards, and progress analytics for self-assessment.	Lin and Wei (2024) and Fitriati and Williyan (2025)

First, participant-derived needs were categorized according to Facione’s (1990) six dimensions of CTS. These needs were grounded in recurring patterns observed in the qualitative data, including learners’ reported difficulties in comprehension monitoring, limited engagement in evaluative reasoning, and minimal use of analytical strategies, as discussed in the preceding sections. Second, these needs were conceptually aligned with AI-mediated pedagogical affordances identified in the literature–technology scan (2020–2025) (see Appendix C) and related pedagogical literature reporting AI- or digitally supported instructional interventions relevant to each CTS dimension. While the identification of learner needs is primarily empirical data-driven, the alignment with specific AI-supported affordances is informed by literature-based synthesis and pedagogical reasoning. This synthesis clarifies that the resulting matrix reflects both observed learner needs and informed design decisions, rather than purely inductive empirical categories. It does not constitute a fully empirically validated framework.

Across studies identified in the scan, AI-mediated feedback, adaptive questioning, and interactive annotation tools were reported to be associated with enhanced engagement, reasoning, and self-regulation in EFL reading contexts (Lee et al., 2024; Crompton et al., 2024; Fitriati and Williyan, 2025). Structured prompts and reflective tasks were reported to scaffold critical reasoning (George and Kumar, 2024), while annotation platforms and feedback dashboards supported collaborative analysis and metacognitive monitoring (Chen et al., 2020; Weidlich et al., 2025). These converging findings are consistent with the CTS-related learner needs identified in the present study. They also illustrate how the Technology–CTS Needs Matrix may inform potential AI-supported interventions across CTS dimensions in blended reading environments. It is important to note that the matrix reflects a conceptual synthesis combining participant-derived empirical findings with literature- and theory-informed design extrapolations, rather than a framework fully validated by empirical data. Therefore, while it provides a structured overview for instructional design, its recommendations should be interpreted as guiding design principles rather than empirically proven prescriptions.

## 6 Conclusion

This study investigated Chinese EFL undergraduates’ needs for developing Critical Thinking Skills within AI-supported blended reading environments. Drawing on McKillip’s Discrepancy Model and Facione’s six-dimensional CTS framework, the study triangulated interview data, AI-mediated interaction records, reflective logs, and relevant literature to identify structural misalignments between learners’ cognitive objectives, instructional design, and AI affordances. The findings suggest that reading engagement remains concentrated at the levels of interpretation and surface-level explanation, while analysis, evaluation, inference, and particularly self-regulation are insufficiently enacted. Although learners recognize the importance of critical engagement, procedural clarity and metacognitive regulation are not systematically integrated with AI-mediated instructional practices, and therefore higher-order reasoning remains underdeveloped. Widespread access to AI tools does not necessarily translate into evaluative or reflective use; instrumental applications such as translation and summarization predominate, suggesting a gap between technological availability and structured cognitive utilization.

Learners consistently articulated the need for explicit CTS modelling, dialogic interaction, and adaptive feedback focused on reasoning quality. These findings suggest that effective CTS development in blended reading contexts may require deliberate alignment among AI affordances, pedagogical design, and cognitive objectives. The Technology–CTS Needs Matrix proposed in this study represents a design-oriented synthesis based on themes manually generated from interviews and reflective logs; literature-identified AI affordances and AI-mediated outputs were included solely for descriptive and contextual support, intended as a guiding framework for instructional design rather than claiming full empirical validation. It outlines how specific CTS dimensions can be supported through coordinated instructional strategies and AI scaffolding, addressing the misalignment between technological tools, instructional structures, and critical thinking development. By articulating a theory-informed, design-oriented, and data-guided model grounded in manually derived thematic insights, with AI outputs and literature consulted for context, the study offers conceptual clarity and a preliminary basis for instructional design for fostering disciplined, self-regulated critical inquiry among Chinese EFL undergraduates. These contributions should be understood as exploratory and design-oriented, pending further empirical validation.

## 7 Limitations and future research directions

This study is based on a single university cohort of Chinese EFL undergraduates, which limits the generalizability of the findings across institutional contexts and learner populations. In addition, reliance on self-reported interviews and reflective logs may introduce subjective bias, as learners’ articulated perceptions do not always fully correspond to enacted cognitive processes. Although AI-mediated interaction records were included for triangulation, the study did not experimentally measure changes in CTS performance, thereby limiting causal interpretation of the identified discrepancies. Therefore, the findings should be interpreted as exploratory and context-specific, rather than as evidence of the effectiveness of any instructional intervention or design model.

Participants’ prior familiarity with AI tools also varied, potentially shaping both their evaluative judgments and their patterns of technological use. The investigation focused primarily on learners’ perspectives; future research incorporating teachers’ viewpoints, classroom observations, and task-level performance data could provide a more ecologically grounded understanding of AI-supported BL practices. Empirical implementation and controlled evaluation of the proposed AI-integrated blended reading framework are necessary to examine its measurable impact on specific CTS dimensions. The proposed Technology–CTS Needs Matrix has not yet been implemented or empirically tested in classroom settings, and its pedagogical effectiveness remains to be examined. Longitudinal studies may further clarify how sustained human–AI interaction may contribute to the procedural internalization of higher-order reasoning over time. Cross-institutional and cross-cultural comparisons would help determine the transferability of the framework across diverse EFL settings, while systematic investigation into teacher AI literacy and pedagogical orchestration could illuminate contextual factors influencing learner outcomes in blended environments.

## Data Availability

The raw data supporting the conclusions of this article will be made available by the authors, without undue reservation.
