# Supplementary material for: Aligning AI affordances with critical thinking skills in Chinese EFL: a discrepancy-based needs analysis for an AI-integrated blended reading module
Source: Front Psychol. 2026 Apr 13;17:1811899. doi: 10.3389/fpsyg.2026.1811899 (PMC13111115; doi:10.3389/fpsyg.2026.1811899)
Supplement: Supplementary file 1 [file Supplementary_file_1.docx]

**Appendix**

**Appendix A** Conceptual design of the AI-assisted reading prototype for expert review

**
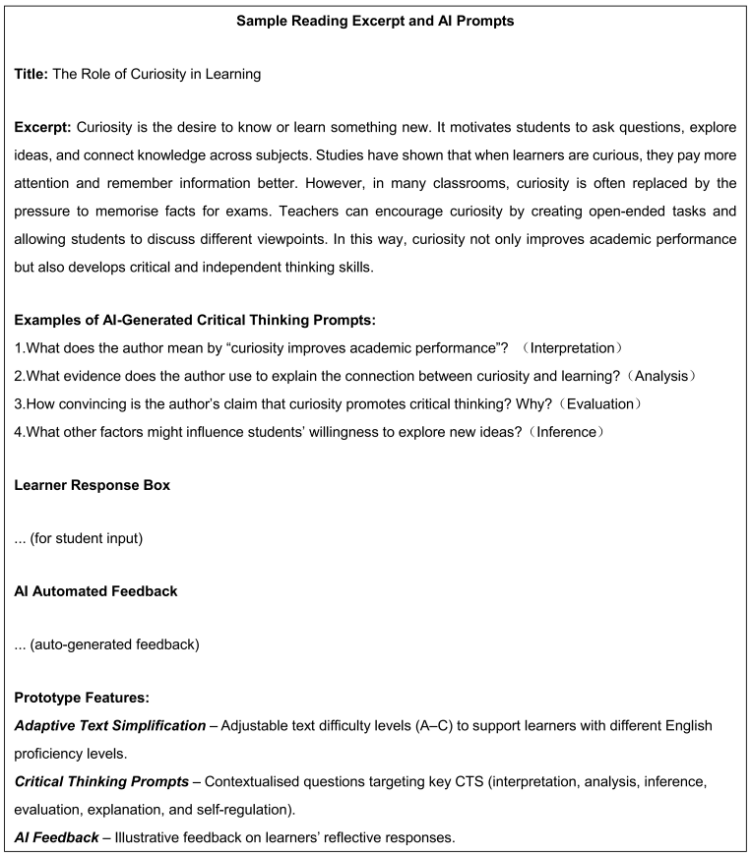
**

**Appendix B** Semi-structured interview guide

| ****Section 1: Current state (RQ1)**** |
| --- |
| 1 How often do you engage in English reading activities in class and online? |
| 2 When you read English articles, what difficulties do you usually face? For example, understanding the language, the ideas, or thinking about the writer’ s opinion? Please give one example. |
| 3 How do you use your critical thinking skills when reading English articles? For example, do you try to understand what the writer means, analyse their ideas, or judge whether you agree with them? Please give one example. |
| 4 What AI or digital tools do you currently use to support English reading, and how effective are they? |
| ****Section 2: Desired state (RQ2)**** |
| 5 Which critical thinking skill would you most like to improve? For example, understand meaning, find connections, judge ideas, draw conclusions, express clearly, and check your thinking. And what kind of support or guidance would help you most? |
| 6 What kind of teacher feedback would help you most in developing critical thinking skills? |
| 7 What AI or digital tool functions would you find most helpful for improving critical thinking skills in reading? Give one example. |
| ****Section 3: Discrepancies and needs (RQ3)**** |
| 8 What are the main differences between your current English reading course and your ideal course with AI support? Please give one or two examples. |
| 9 What challenges or concerns do you have about using AI tools for critical thinking skills in reading? |
| 10 What kinds of help or support from your teacher, classmates, or AI tools would be most useful for you in learning reading through both online and face-to-face ways? Please give one example. |
| ****Section 4: Implications for module design (RQ4)**** |
| 11 What specific features or functions would you like an AI-supported blended reading module to include? |
| 12 How do you think online AI activities and face-to-face classes should work together to help you think more critically when reading? For example, what parts are better online, and what parts are better in class? |
| 13 How could the AI-assisted reading module prototype you experienced help you improve your critical thinking skills when reading English texts? |

**Appendix C** Summary of the literature–technology scan (2020–2025)

| **No** | **Authors**  **＆Year** | **Focus/**  **Context** | **Technology/**  **AI tool (s)** | **Key Findings** | **Implication for the Study** |
| --- | --- | --- | --- | --- | --- |
| 1 | Cao & Phongsatha (2025) | Quasi-experiment on a FLIT-based BL model for Business English at a Chinese university. | FLIT platform integrating speech recognition, adaptive response scoring, real-time analytics, and personalized feedback loops. | Substantial improvements were observed across all four language skills, accompanied by high levels of cognitive and behavioral engagement. | Highlights the value of AI-driven, feedback-rich BL for enhancing engagement and proficiency, reinforcing the rationale for adaptive integration in the proposed AI-supported reading module. |
| 2 | Chen et al. (2020) | Quasi-experiment on a gamified Web-based Collaborative Reading Annotation System (WCRAS) for learners in Taiwan. | Gamified online annotation platform with levels, leaderboards, and scaffolds. | Gamification increased interaction and participation. | Highlights the need to align engagement strategies with cognitive objectives in AI-supported blended reading. |
| 3 | Crompton et al. (2024) | PRISMA review of AI affordances and challenges in 42 studies across K-12, higher education, and adult ELT/L. | Chatbots (ChatGPT), voice assistants (Alexa), grammar checkers (Grammarly), speech recognition, translation, VR/AI tutors. | AI-assisted language learning shows strong potential for enhancing skill development, pedagogical support, and learner self-regulation across contexts. | Provides an integrative perspective on AI’s educational affordances, informing the design of literacy-oriented, critically and ethically grounded AI integration in blended EFL reading. |
| 4 | Er et al. (2025) | Experimental study comparing AI-generated and instructor feedback in higher education learning tasks. | AI feedback system powered by a Large Language Model (LLM) providing rubric-aligned formative comments. | Instructor feedback yielded higher learning gains, while AI-generated feedback offered consistent formative support. | Emphasizes refining AI feedback to better support higher-order learning and adaptive integration in blended reading design. |
| 5 | Fitriati & Williyan (2025) | PRISMA review of 15 studies on generative AI and LLM use for developing EFL learners’ critical thinking. | ChatGPT, SummarizeBot, ParagraphAI, speech bots. | Most studies report that AI-mediated feedback, summarization, and argument mapping contribute to developing learners’ critical thinking in reading and reasoning contexts. | Highlights the growing potential of generative AI to scaffold critical thinking and promote human-AI synergy and AI-literacy development in blended reading contexts. |
| 6 | George & Kumar (2024) | Conceptual exploration of integrating generative AI to foster critical thinking in higher education skill-based or reading-related modules. | ChatGPT 3.5 and AI chatbots designed with the Paul-Elder framework and systems-thinking prompts. | Structured AI prompts and reflective tasks scaffolded learners’ critical reasoning and engagement in blended reading contexts. | Demonstrates how pedagogically framed AI use can humanize learning and scaffold critical thinking, informing the design of AI-integrated blended reading modules. |
| 7 | Liu et al. (2024a) | Mixed-method study on Chinese EFL learners’ acceptance and use of GPT chatbots for informal AI-supported language learning. | ChatGPT, Bing Chat, and Ernie Bot with voice and tutoring features. | Learners’ adoption was influenced by perceived usefulness, accessibility, and prompt-crafting skills, fostering creative informal learning practices. | Highlights the importance of embedding AI-literacy and prompt-training into blended reading pedagogy to bridge formal and informal learning practices. |
| 8 | Liu et al. (2024b) | Quantitative study on Chinese EFL students’ motivation and engagement in AI-assisted informal English learning. | Generative AI tools (e.g., ChatGPT) supporting reflective dialogue and critical reading engagement. | Learners’ enjoyment and ideal L2 self promoted active engagement and persistence in AI-mediated learning, suggesting affective factors underpin learners’ readiness for AI-supported blended reading. | Indicates the need for AI-integrated blended reading designs that align affective engagement with cognitive and reflective tasks to foster critical thinking growth. |
| 9 | Teng et al. (2024) | Study on teaching, cognitive, and social presence in technology-enhanced reading circles within blended EFL learning. | Technology-enhanced reading-circle platform integrating discussion, annotation, and feedback functions. | Teaching and cognitive presence significantly influenced learners’ engagement and attitudes toward blended reading. | Reinforces the pedagogical importance of strengthening cognitive and teaching presence through AI-mediated scaffolding and interaction design in blended reading contexts. |
| 10 | Weidlich et al. (2025) | Study on AI-driven highly informative feedback (HIF) in higher education reading contexts. | AI-driven learning analytics feedback systems. | HIF enhanced learners’ understanding of progress, self-regulation, and motivation, contributing to greater metacognitive awareness and critical engagement. | Underscores the importance of designing AI-supported blended reading modules that embed analytic, personalized feedback to foster learners’ critical thinking, reflective reading, and self-regulated learning. |

**Appendix D** Screenshot of a sample interaction report from the AI-assisted reading prototype

**
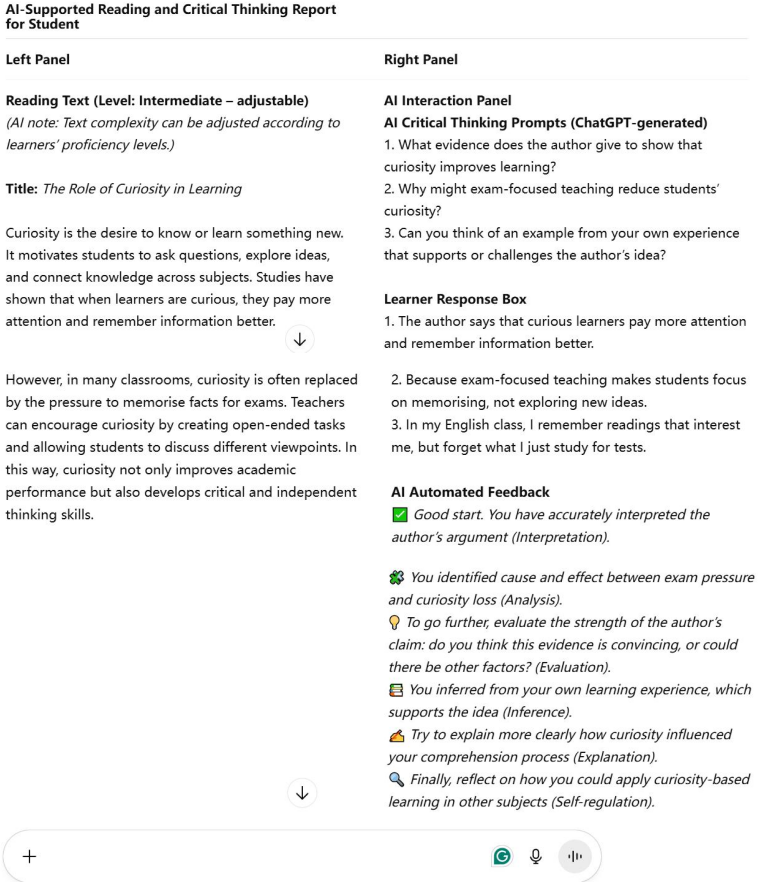
**

**Appendix E** Illustrative interview extracts with coding, AI NLP key phrases, and sentiment

| **Deductive Coding** | | | **AI-assisted NLP Output** | |
| --- | --- | --- | --- | --- |
| **Main Code** | **Subcode** | **Participant Extract** | **Key Phrases** | **Sentiment** |
| Evaluation | Current  Ideal  Discrepancy | “I usually just believe the answers in the textbook and rarely question them.”  “It’d be great if the AI could let me compare different viewpoints.”  “I know I’m supposed to think critically, but I’m not sure what the standard actually is.” | “believe answers”, “textbook”, “rarely question”;  “compare viewpoints”, “AI support”, “great”;  “think critically”, “not sure”, “standard”. | neutral/slightly passive;  positive/engaged;  neutral/slightly passive. |
| **Inductive Coding** | | | **AI-assisted NLP Output** | |
| **Theme** | | **Participant Extract** | **Key Phrases** | **Sentiment** |
| Affective response to AI | | “At first I thought the AI was too ‘smart’, and I was a bit scared of giving the wrong answers. Later I realized it’s actually quite patient.” | “AI too smart”, “scared of wrong answers”, “quite patient” | neutral → positive / slightly engaged. |
